# Plasmonic Metal–Phenolic Network Nanoprobes for Multiplex Dual-Mode Immunophenotyping

**DOI:** 10.1021/acs.nanolett.5c05298

**Published:** 2025-12-16

**Authors:** Lara González-Cabaleiro, Zhixing Lin, Lorena Vázquez-Iglesias, Soraia Fernandes, Sergio Rodal-Cedeira, Gustavo Bodelón, Jorge Pérez-Juste, Frank Caruso, Isabel Pastoriza-Santos

**Affiliations:** † CINBIO, 16784 Universidade de Vigo, Campus Universitario As Lagoas, Marcosende, 36310 Vigo, Spain; ‡ Departamento de Química Física, Universidade de Vigo, Campus Universitario As Lagoas, Marcosende, 36310 Vigo, Spain; § Department of Chemical Engineering, 2281 The University of Melbourne, Parkville, Victoria 3010, Australia; ∥ Department of Chemical and Petroleum Engineering, Research and Innovation Center for Graphene and 2D Materials, 105955 Khalifa University, Abu Dhabi 127788, United Arab Emirates; ⊥ International Clinical Research Center, St. Anne’s University Hospital, 65691 Brno, Czech Republic; # Departamento de Biología Funcional y Ciencias de la Salud, Universidade de Vigo, Campus Universitario As Lagoas, Marcosende, 36310 Vigo, Spain

**Keywords:** metal−phenolic network, SERS tags, dual mode, cell targeting

## Abstract

Accurate multiplexed immunophenotyping is essential for understanding cellular heterogeneity. Dual-mode detection strategies, such as surface-enhanced Raman scattering (SERS)–fluorescence, offer synergistic advantages and cross-validation to reduce false positives. We present a method for fabricating dual-mode nanoprobes using metal–phenolic networks (MPNs) as functional coatings on hollow plasmonic nanocapsules. Unlike conventional silica shells, MPNs enable the one-step conjugation of biorecognition elements via metal–phenolic coordination, integrating distinct Raman reporters and fluorescently labeled antibodies within a single probe. These plasmonic MPN nanoprobes achieved multiplex detection of the epidermal growth factor receptor and CD44 in cultured cells using SERS and flow cytometry. Application to mixed HER14 and HEK-293 cultures revealed population distributions of ∼63% HER14 and ∼37% HEK-293 as determined by SERS, corroborated by flow cytometry. This work highlights the potential of dual-mode plasmonic MPNs for accurate, scalable immunophenotyping, offering a robust platform for targeted cell imaging and diagnostics.

## Graphical abstract

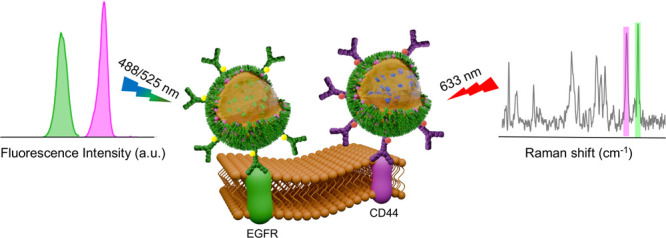

Accurate and early detection of diseases is essential for improving diagnostics and optimizing therapeutic strategies. However, conventional detection techniques often fall short in sensitivity, specificity, and multiplexing capabilities, underscoring the need for more advanced analytical platforms.^1^ Multimodal detection integrates complementary analytical techniques to provide reliable, cross-validated results.^1–3^ Each technique contributes specific capabilities, such as high sensitivity, specificity, and the ability to analyze different sample properties (e.g., chemical composition, structure, and spatial distribution).^3,4^ Using this synergistic approach, it is possible to overcome the limitations of individual techniques, such as background noise, interference, and a limited detection range. Surface-enhanced Raman scattering (SERS) has been combined with orthogonal methods,^5–7^ including electrochemistry, mass spectrometry, and fluorescence, to enhance robustness and information depth.^3,8^ In particular, dual-mode SERS–fluorescence systems unite the molecular specificity and multiplexing power of SERS with the sensitivity and imaging speed of fluorescence, enabling accurate, multiple biomarker detection in complex biological samples.^3^

Immunophenotyping is a cornerstone analytical strategy that uses specific antibodies for identifying and classifying cells based on the expression of surface and intracellular proteins as target biomarkers.^9,10^ It is widely used in both clinical and research settings, as it enables high-dimensional profiling of cellular heterogeneity, lineage, and phenotypic states in health and disease.^11^ Widely applied in clinical diagnostics, infectious disease monitoring, and personalized treatments, it also underpins pharmacology, immunology, regenerative medicine, and stem cell research by fingerprinting cellular proteomes with precision.^12–14^

Plasmonic nanoparticles, particularly Au and Ag, are ideal platforms for multimodal probes owing to their tunable optical properties and surface chemistry.^15^ Previous studies have reported the use of these metallic nanoparticles as dual-mode SERS–fluorescence strategies for the detection of proteins and cellular imaging. Traditional dual-mode SERS–fluorescence strategies often use silica coatings^16–19^ as they facilitate the immobilization of Raman reporters and fluorophores, aptamer bioconjugation, and enhanced colloidal stability for protein detection and cellular imaging applications.^20,21^ However, their fabrication typically involves complex, multistep protocols and precise control of shell thickness to avoid fluorescence quenching and optimize their performance.^20,22–24^ To address these limitations,^25–27^ we propose the use of metal–phenolic networks (MPNs) as a simplified and reliable platform to fabricate bimodal SERS–fluorescence nanoprobes with encoding capacity, high colloidal stability, and antibody functionalization capability. MPNs are supramolecular assemblies that self-assemble through the coordination of phenolic compounds [e.g., tannic acid (TA), quercetin, and epigallocatechin gallate] with metal ions (such as Fe^3+^, Cu^2+^, and Zn^2+^).^28^ These networks have gained attention in biomedical and biological applications owing to their tunable physicochemical properties, biocompatibility, and ease of assembly.^29^ Notably, MPNs support the incorporation of fluorescent compounds^30^ and enable the one-step conjugation of proteins, including antibodies, via coordination,^31,32^ streamlining the fabrication of functional nanoprobes. Table S1 of the Supporting Information summarizes the dual-mode SERS–fluorescence strategies for protein detection and cellular imaging.

Herein, we demonstrate that plasmonic MPN (P-MPN) nanoprobes, based on hollow plasmonic nanocapsules, enable the simultaneous detection and imaging of epidermal growth factor receptor (EGFR) and CD44 cell surface receptors in cultured cells via SERS and fluorescence flow cytometry (Scheme 1). The simplicity of MPN coating of metallic nanoparticles and one-step antibody conjugation, combined with the performance of the nanoprobes in SERS detection, makes this approach suitable for the fabrication of dual-mode SERS–fluorescence nanoprobes with potential in cell immunophenotyping, targeted imaging, antigen immunoassays, and other biomolecular recognition-based analytical applications.

**Scheme 1:**
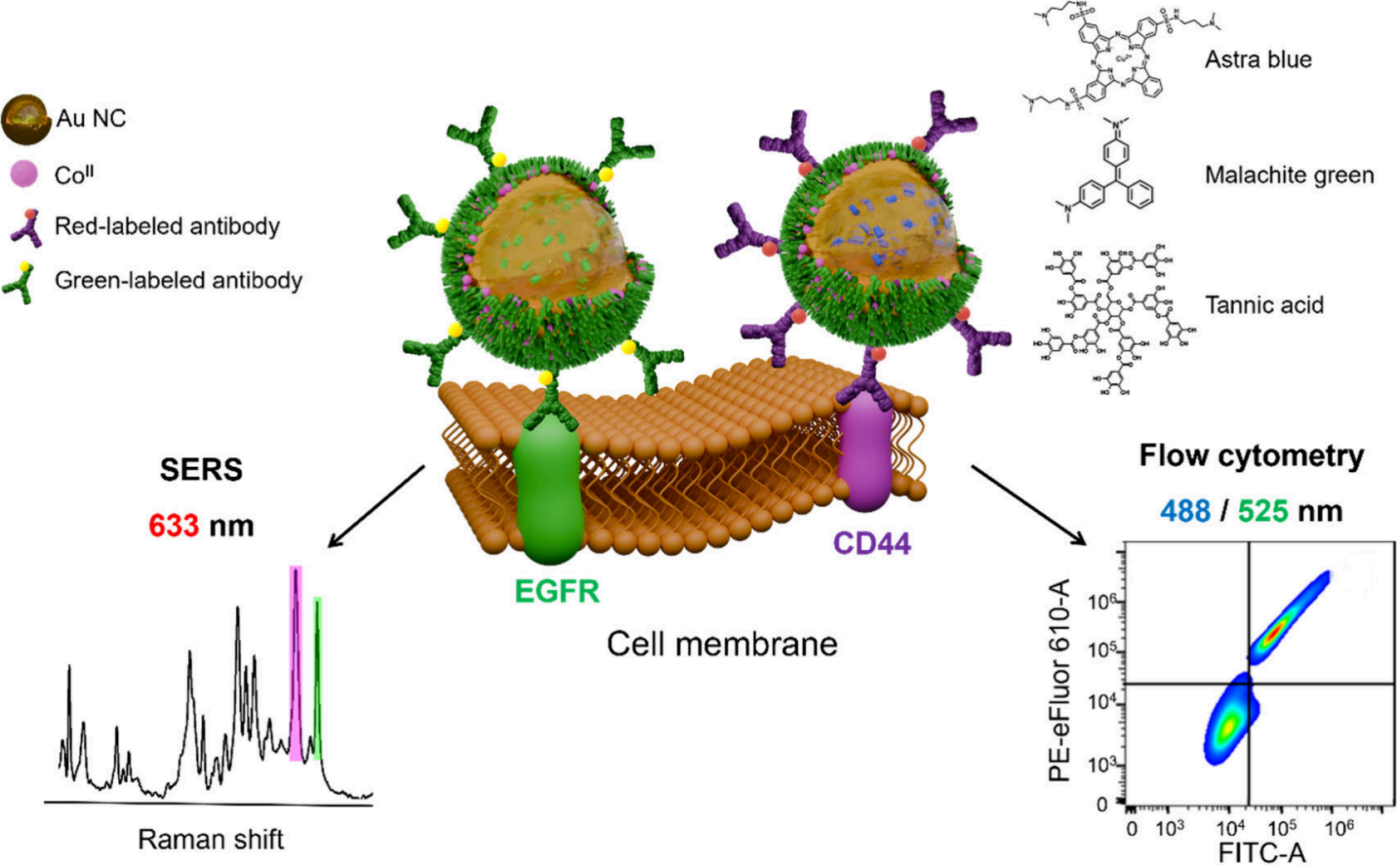
Schematic Representation of the Multiplex Targeting of EGFR and CD44 Cell Surface Receptors by SERS and Fluorescence Flow Cytometry^a^

a The dual-mode hollow P-MPN probes were functionalized with MG or AB as Raman reporters and conjugated with FITC-labeled anti-EGFR (green-labeled antibody) or PE–eFluor 610-labeled anti-CD44 (red-labeled antibody), respectively, starting from hollow gold nanocapsules (Au NCs). The SERS excitation laser line was a 633 nm laser line, and the Raman signals from MG and AB are indicated in green and pink, respectively. Immunophenotyping by flow cytometry was performed employing 488 and 525 nm laser lines to detect FITC- and PE–eFluor 610-labeled nanoprobes, respectively.

## Synthesis of the Dual-Mode Nanoprobes

For the immunophenotyping of EGFR and CD44, we employed dual-mode nanoprobes based on SERS-encoded hollow Au nanocapsules coated with MPNs and biofunctionalized with fluorescently labeled antibodies. To target EGFR, nanocapsules encoded with malachite green (MG) and conjugated with fluorescein isothiocyanate (FITC)-labeled anti-EGFR antibodies were synthesized (AuMG@MPN-anti_EGFR_@FITC, hereafter referred to as P-MPN_EGFR_). For CD44 detection, nanocapsules encoded with astra blue (AB) were functionalized with phycoerythrin–eFluor 610 (PE–eFluor 610)-labeled anti-CD44 antibodies (AuAB@MPN-anti_CD44_@PE–eFluor 610, hereafter referred to as P-MPN_CD44_).

The fabrication process involved a two-step protocol: (i) synthesis of dye-encoded hollow Au nanocapsules, as previously reported,^33^ and (ii) MPN coating and antibody conjugation via a single-pot coordination-driven assembly (Figure 1A).^34,35^ Briefly, a 1:1 mixture of Co^II^ ions and TA, along with the respective antibody, was added to the encoded nanocapsules, followed by pH adjustment to trigger MPN film formation through metal–polyphenol coordination. TA was selected due to its higher stability and biocompatibility than other phenols. Co^II^ ions were selected, as Co^II^–TA MPNs showed a high efficiency in antibody-mediated particle targeting.^34^ The enhanced antigen binding was attributed to the higher concentration of solvent-exposed Co^II^ promoting coordination binding with the histidine-rich domain of the antibody Fc region.^36^ The MPN coating induced a red shift in the surface plasmon resonance of the Au nanocapsules due to their higher refractive index than water and increased low-wavelength absorbance from Co^II^–TA charge-transfer interactions (Figure 1B). Transmission electron microscopy (TEM) confirmed the MPN deposition with an average thickness of 2–3 nm (Figure 1C and Figure S1).

**Figure 1:**
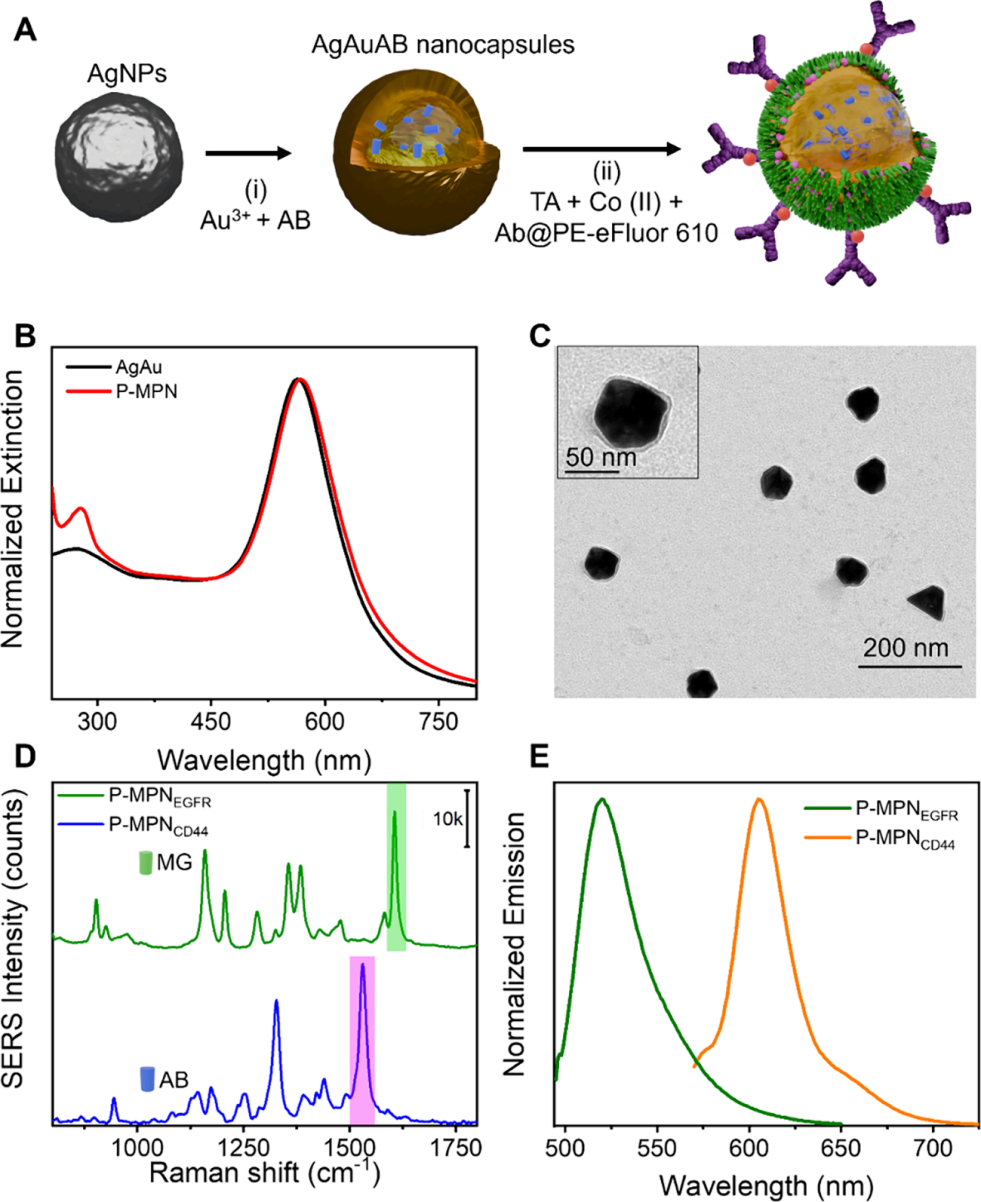
(A) Schematic representation of the synthesis of dual-mode probes: (i) synthesis of Au hollow nanocapsules encoded with a Raman reporter (e.g., AB) and (ii) MPN coating and biofunctionalization with labeled antibodies (Abs). (B) UV–vis–near-infrared extinction spectra of encoded hollow Au nanocapsules before (black curve) and after (red curve) MPN coating and antibody labeling. (C) TEM image of the P-MPN–Ab nanoprobes. (Inset) Higher magnification TEM image of a single nanoparticle. (D) SERS spectra of the dual-mode probes encoded with MG (green) or AB (pink). The green- and pink-shaded regions indicate characteristic Raman peaks of MG (1617 cm^–1^) and AB (1540 cm^–1^), respectively. (E) Fluorescence spectra of FITC- and PE–eFluor 610-labeled antibodies in the P-MPN nanoprobes.

Dual-mode detection capabilities were validated through SERS and fluorescence measurements. As shown in Figure 1D, the nanoprobes exhibited distinct Raman spectral signatures corresponding to their encoded dyes (MG or AB), with shaded regions indicating diagnostic peaks used in downstream analysis. Fluorescence spectroscopy confirmed the presence of FITC (anti-EGFR) and PE–eFluor 610 (anti-CD44) labels (Figure 1E). Antibody conjugation was independently confirmed via a dot blot analysis (Figure S2).

## EGFR and CD44 Biomarker Immunophenotyping

To assess the performance and specificity of the dual-mode nanoprobes for cell immunophenotyping, HER14 cells (EGFR/CD44 overexpressing) and HEK-293 cells as a control (low EGFR/CD44) were used (Figure S3). Accordingly, P-MPN_EGFR_ and P-MPN_CD44_ nanoprobes were incubated separately with HER14 and HEK-293 cells, and specific binding was assessed by SERS.

For EGFR detection, HER14 and HEK-293 cells were incubated with P-MPN_EGFR_ probes, washed to remove unbound nanoprobes, and analyzed by SERS (Figure 2).^37^ At least 100 cells were measured, with three SERS point mappings acquired per cell (Figure 2A). A cell was classified as positive if two out of three points exceeded the background, defined as points outside the cells (Figure 2C). SERS mapping at 1617 cm^–1^ (MG signature) revealed the presence of EGFR in 93% of HER14 cells and only 8% of HEK-293 (Figure 2A and B), consistent with the EGFR expression in HER14 and HEK-293 cell lines (Figure S3). Spatial correlation between the Raman signal and cellular location further validated probe specificity, as a negligible signal was detected in the surrounding areas (Figure 2B). Complementary analysis via flow cytometry detected FITC fluorescence from the antibody-labeled nanoprobes, yielding comparable results: 97.5% EGFR-positive HER14 versus 2.36% HEK-293 (Figure 2D). A minimal signal in the control group likely reflects low expression levels or non-specific interactions. Concordance between SERS and flow cytometry underscores the reliability and robustness of the dual-mode nanoprobes, reinforcing their utility for precise and high-confidence biomarker profiling.

**Figure 2:**
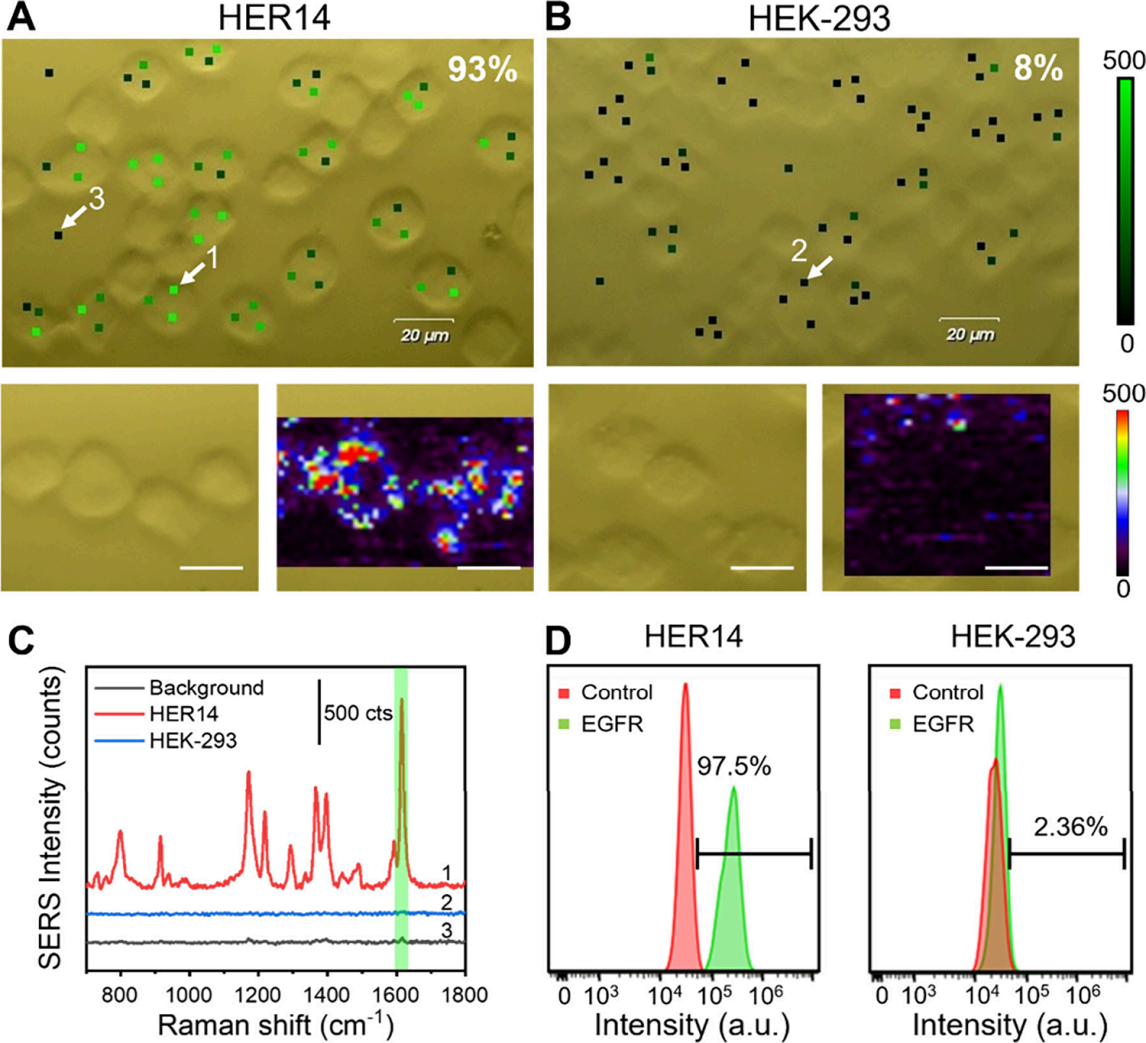
Dual-mode detection of EGFR in HER14 and HEK-293 cells using P-MPN_EGFR_ probes. (A and B) Bright-field images and SERS mappings of (A) HER14 and (B) HEK-293 cells. The white arrows indicate the points whose spectra are represented in panel C. SERS spectra were acquired with a 633 nm laser line, a 50× objective, and a laser power of 5 mW. Scale bars are 20 μm. (C) Representative SERS spectra recorded in HER14 (red spectrum) and HEK-293 (blue spectrum) cells and the background (black spectrum). The green band highlights the Raman peak of MG used for SERS mapping. (D) Flow cytometry analysis of the binding of the P-MPN_EGFR_ probe to HER14 or HEK-293 cells. Flow cytometry was performed by using a 488 nm laser line.

For CD44 detection, HER14 and HEK-293 cells were incubated with the P-MPN_CD44_ nanoprobe and analyzed by SERS at 1540 cm^–1^ (AB signature). CD44 expression was detected in 90% of HER14 cells versus 5.5% of HEK-293, consistent with the flow cytometry analysis (Figure S3). Consistent with the EGFR results, SERS maps also demonstrated a strong spatial correlation between the signal and cell location (Figure 3A and B). Representative SERS spectra (Figure 3C) further illustrated the distinction between HER14 (red spectrum) and HEK-293 (blue spectrum) in comparison to the background spectrum (black spectrum). In this case, the background slightly exceeded the HEK-293 signal in some points. This overlap may account for the lower detection rate in HER14 cells, likely due to the relatively reduced SERS efficiency of AB compared to that of MG.

**Figure 3:**
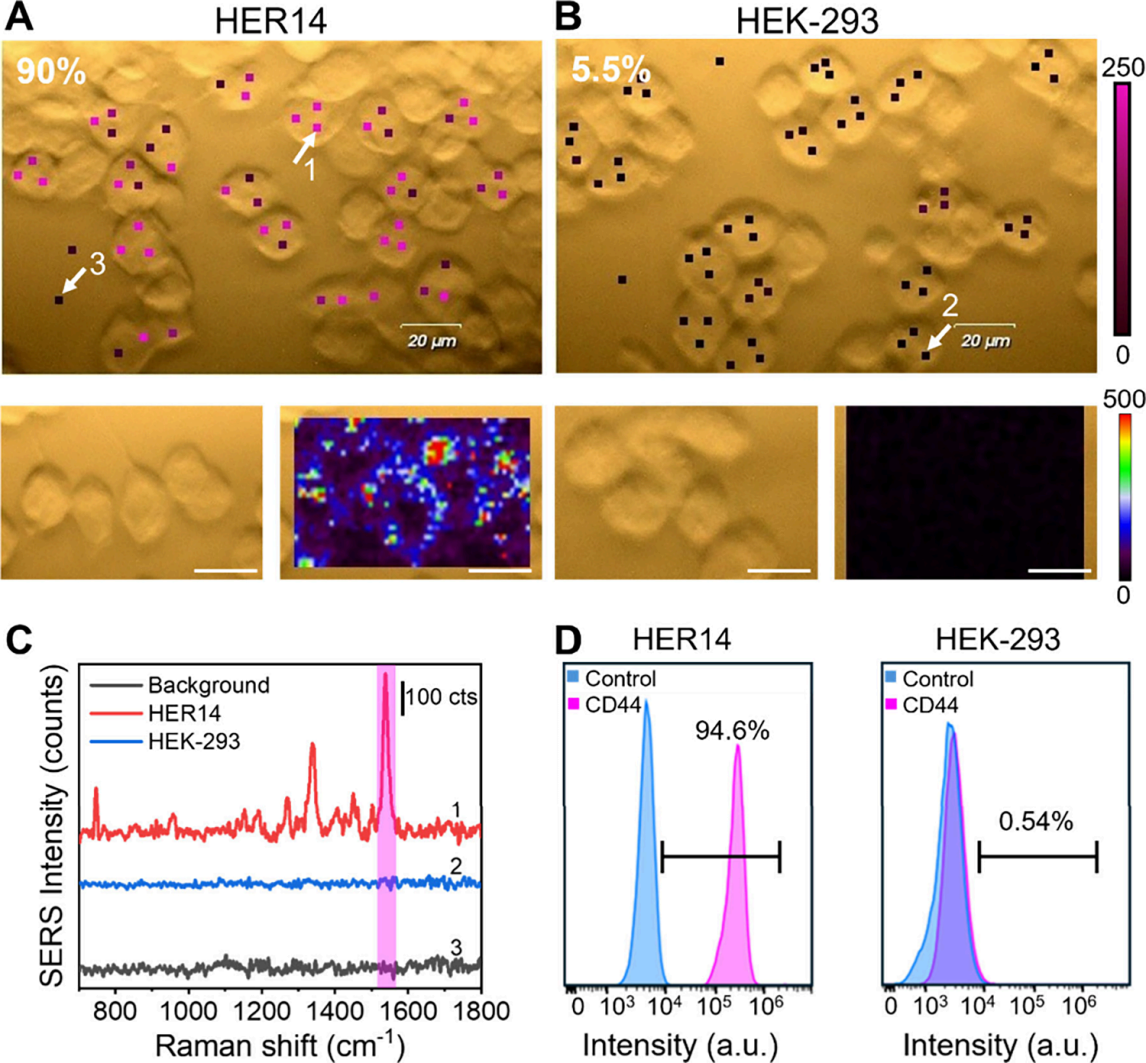
Dual-mode detection of CD44 in HER14 and HEK-293 cell cultures with P-MPN_CD44_ dual probe. (A and B) Bright-field images and SERS mappings of (A) HER14 and (B) HEK-293 cells. The white arrows indicate the points whose spectra are represented in panel C. SERS spectra were acquired with a 633 nm laser line, a 50× objective, and a laser power of 5 mW. Scale bars are 20 μm. (C) Representative SERS spectra recorded in HER14 (red spectrum) and HEK-293 (blue spectrum) cells and the background (black spectrum). The pink band highlights the Raman peak of AB used for SERS mappings. (D) Flow cytometry analysis of the binding of P-MPN_CD44_ to HER14 or HEK-293 cells. Flow cytometry was performed by using a 525 nm laser line.

Flow cytometry analysis (Figure 3D and Figure S4) corroborated these findings, detecting CD44 in 94.6% of HER14 cells and 0.54% of HEK-293 cells, closely matching the SERS-derived percentages with only a minor deviation observed for each cell line. These results further highlight the enhanced specificity and robustness of this dual-mode detection strategy.

## Multiplex EGFR and CD44 Immunophenotyping in Mixed Cell Cultures

We next examined the nanoprobes’ SERS performance in mixed cell cultures. The P-MPN_EGFR_ and P-MPN_CD44_ probes were incubated with co-cultures of HER14 and HEK-293 cells. Statistical SERS analysis revealed a cell population consisting of approximately 63% HER14 and 37% HEK-293 cells (Figure 4). In this assay, four measurement points per cell were acquired and a cell was classified as positive when at least three out of four spectra exhibited signal intensities above the background threshold. Representative SERS spectra (Figure 4C) from cells 1 and 2 (Figure 4A and B) displayed MG and AB peaks, indicating the EGFR and CD44 presence and identifying them as HER14 cells. In contrast, cells 3 and 4 displayed no Raman signals, consistent with the HEK-293 phenotype (Figure 4C). Among HER14 cells, EGFR signals were more prominent than CD44, likely reflecting biological variability in protein expression across the population and the higher SERS efficiency of MG over AB under 633 nm excitation.

**Figure 4:**
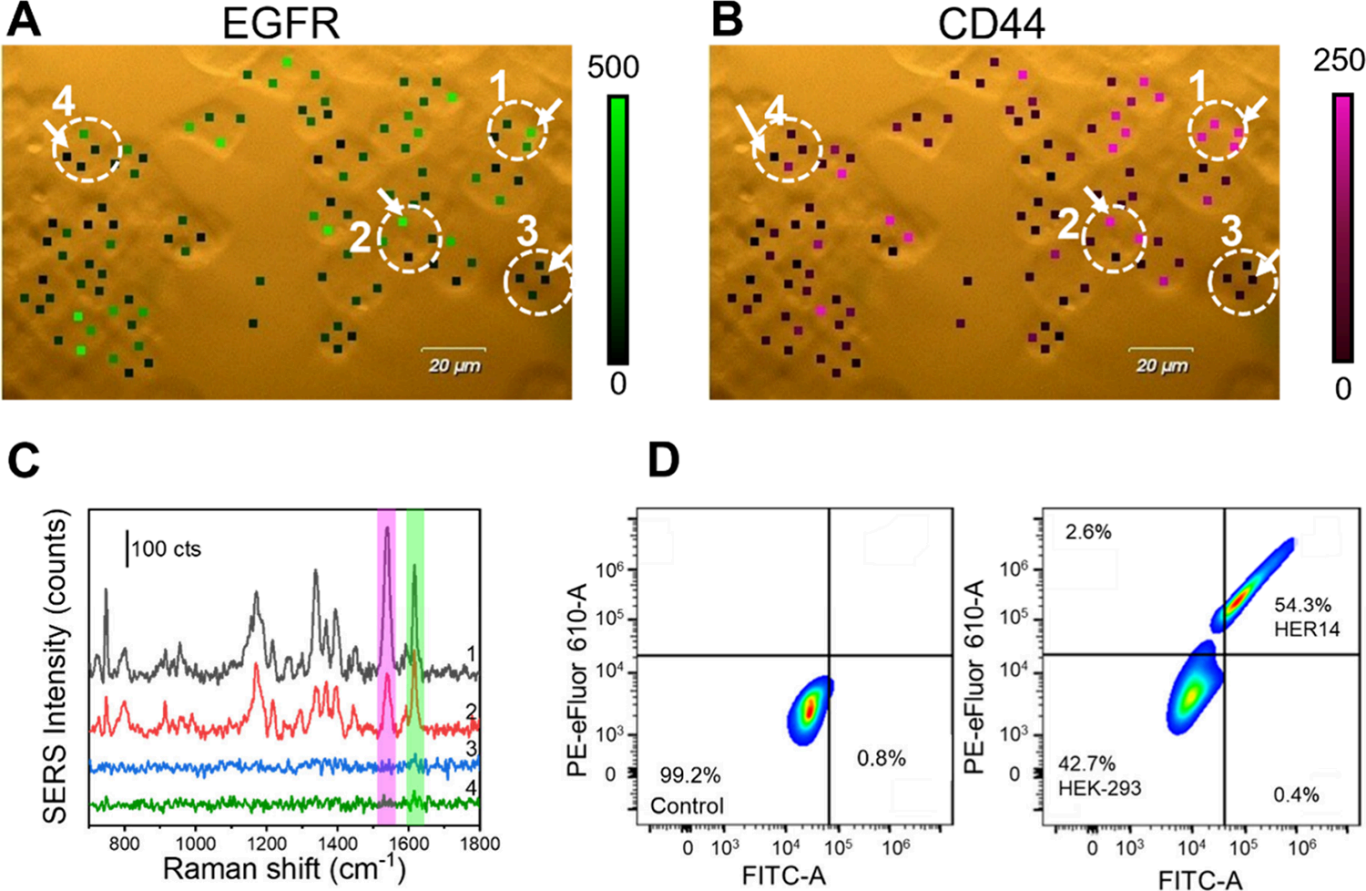
SERS detection of EGFR and CD44 in mixed cultures of HER14 and HEK-293 cells using dual-mode P-MPN_EGFR_ and P-MPN_CD44_ probes. (A and B) Bright-field images of mixed HER14 and HEK-293 cells and single-point SERS mappings recorded at 1617 cm^–1^ (MG, map in panel A) and 1540 cm^–1^ (AB, map in panel B) for the detection of EGFR and CD44, respectively. Scale bars are 20 μm, with a 633 nm laser excitation line, 50× objective, and 5 mW. (C) Representative SERS spectra from the cells indicated with a white dashed circle and numbered 1–4 in the bright-field images in panels A and B. (D) Flow cytometry diagrams of the binding of EGFR (FITC-labeled) and CD44 (PE–eFluor 610-labeled) in a mixed culture (right) plot. Flow cytometry was performed by using 488 and 525 nm laser lines.

Following nanoprobe binding, the population distribution assessed by flow cytometry (Figure 4D and Figure S5) revealed 54.3% HER14 cells (double-positive, upper right quadrant), 42.7% HEK-293 cells (double-negative, lower left quadrant), and negligible single-positive cells (approximately 3% single-positive cells, i.e., 2.6% for CD44 and 0.4% for EGFR), likely reflecting heterogeneity in biomarker expression within the cell lines. These values closely align with those obtained from the SERS-based analysis. The slight overrepresentation of HER14 cells observed in both data sets may stem from the differential growth or adherence efficiencies under mixed culture conditions.

Collectively, the results demonstrate that the P-MPN nanoprobes enable robust simultaneous detection of EGFR and CD44 receptors, combining the molecular-specific information from SERS with the high-throughput and fast analysis of fluorescence cytometry. Importantly, the approach based on MPNs provides a straightforward, robust, and reliable strategy for the fabrication of dual-mode SERS–fluorescence nanoprobes amenable to cellular immunophenotyping and biosensing proteins as antigens.

A general strategy to fabricate dual-mode SERS–fluorescence P-MPN probes were demonstrated. The MPN shell played two main key roles: enhancing the colloidal stability of the Raman-encoded Au nanocapsules and facilitating bioconjugation with fluorescently labeled antibodies. The functional activity and specificity of the dual-mode P-MPN probes was demonstrated for the detection of EGFR and CD44 biomarkers in single and mixed cell cultures, showcasing the potential of these optical nanoprobes for SERS and fluorescence analysis. This versatile modular assembly approach can be employed as a universal system for functionalizing MPN-based materials, useful in biosensing and biotechnological applications with higher sensitivity and specificity, such as portable point-of-care testing, lab-on-a-chip devices, and advanced imaging tools.

## Supplementary Material



## Data Availability

Additional data reported in this paper can be provided by the corresponding authors upon reasonable request.

## Notes

Additional details on the sample preparation process, characterization, and supporting data, including TEM images of Au hollow capsules and P-MPN, dot blot analysis of P-MPN, flow cytometry for cell phenotyping and cell binding, and dual-mode SERS–fluorescence strategies for the optical detection of proteins and cellular imaging (Table S1) (PDF)
