# Supplementary material for: Plasmonic Metal–Phenolic Network Nanoprobes for Multiplex Dual-Mode Immunophenotyping
Source: Nano Lett. 2025 Dec 16;25(51):17911–7. doi: 10.1021/acs.nanolett.5c05298 (PMC12751108; doi:10.1021/acs.nanolett.5c05298)
Supplement: Supplementary file 1 [file nl5c05298_si_001.pdf]

Supporting Information

Plasmonic Metal–Phenolic Network Nanoprobes for Multiplex Dual-Mode Immunophenotyping

Lara González-Cabaleiro,[a,b] Zhixing Lin,[c,d] Lorena Vázquez-Iglesias,[a] Soraia Fernandes,[c,e] Sergio Rodal-Cedeira,[a,b] Gustavo Bodelón,[a,f] Jorge Pérez-Juste,[a,b] Frank Caruso,*[c] and Isabel Pastoriza-Santos*[a,b]

[a] CINBIO, Universidade de Vigo, Campus Universitario As Lagoas, Marcosende, 36310 Vigo, Spain

[b] Departamento de Química Física, Universidade de Vigo, Campus Universitario As Lagoas, Marcosende, 36310 Vigo, Spain

[c] Department of Chemical Engineering, The University of Melbourne, Parkville, Victoria 3010, Australia

[d] Department of Chemical and Petroleum Engineering, Research and Innovation Center for Graphene and 2D Materials, Khalifa University, Abu Dhabi 127788, United Arab Emirates.

[e] International Clinical Research Center, St. Anne's University Hospital, 65691 Brno, Czech Republic

[f] Departamento de Biología Funcional y Ciencias de la Salud, Universidade de Vigo, Campus Universitario As Lagoas, Marcosende, 36310 Vigo, Spain

*Corresponding authors. Email: fcaruso@unimelb.edu.au (F.C.); pastoriza@uvigo.gal (I.P.-S.)

Table S1. Summary of dual-mode SERS-fluorescence strategies for the optical detection of proteins and cellular imaging.

Plasmonic nanoparticle	Raman reporter	Fluorescence	Coating	Targeting ligand	Cell	Reference
Ag NS	3,4-DCT, 4-CBT, 4-BBT, 4-FBT	RITC	Silica	CD44 antibody	MDA-MB-231	[1]
Ag NS	4-MPY	FMN	Silica	None	MCF-7	[2]
Ag NS	4-ATP	CdS QDs	Silica	Anti-IgG	None	[3]
Au@Ag NR	4-MBA, DTNB	CdTe QDs	Silica	Anti-IgG	None	[4]
Au NS	N.D.	Alexa Fluor 488, Alexa Fluor 647	Silica	Her2/CD44 antibodies	MDA-MB-231, BT-549	[5]
Ag NS	4-MBA	RBITC	Silica	T7/c(RGDyC)	HeLa	[6]
Au@Ag NR	4-MBA	CdTe QDs	Silica	Folic acid	HeLa	[7]
Au NR	DTNB	RhBITC	Silica	Folic acid	HeLa	[8]
Au NS	4-MBA	CPN	Silica	GP3/CA153 antibodies	Tumor cell lines	[9]
Au NS	Rubpy, MGITC	RuITC, FITC	Silica	CD24/CD44 antibodies	MDA-MB-231	[10]
Ag NS	CV, MGITC, Rh6G, DTDC, DTTC	UCNP	Silica	None	MCF-7	[11]
Au NR	Cy5.5, Cy7, Dylight747, Dylight780	Dylight780	Silica, PEG	None	Tumor cell lines	[12]
Au HNP	IR780, R6G	FITC	PDA, BSA	EpCAM/EGFR antibodies	Breast cancer cell lines	[13]
Au NS	PDDA	CdSe@ZnS	PEG	Muc1 aptamer	HepG-2, MCF-7	[14]
Au NR	4-MBA	$CaMoO_4:Eu^{3+}$	PEG	GPR120 antibody	HEK293	[15]
Au@Ag NR	4-ATP	FITC	DNA	PTK-7 aptamer	HeLa	[16]
P-MPN_{EGFR}	MG	FITC	MPN	EGFR	HER14, HEK-293	This work
P-MPN_{CD44}	AB	PE-eFluor 610	MPN	CD44	HER14, HEK-293	This work

NS, nanostar; DCT, dichlorobenzaldehyde; CBT, chlorobenzenethiol; BBT, bromobenzenethiol; FBT, fluorobenzenethiol; RITC, rhodamine isothiocyanate; MPY, mercaptopyridine; FMN, flavin mononucleotide; MCF, Michigan Cancer Foundation; ATP, aminothiophenol; QDs, quantum dots; IgG, immunoglobulin G; NR, nanorod; MBA, mercaptobenzoic acid; DTNB, 2-nitrobenzoic acid; N.D., non-described; RBITC, rhodamine B isothiocyanate; RGDyC, cyclo(Arg-Gly-Asp-D-Tyr-Cys); RhBITC, rhodamine B isothiocyanate; CPN, conjugated polymer nanoparticle; Rubpy, tris(2,20-bipyridyl)ruthenium(II) chloride; MGITC, malachite green isothiocyanate; RuITC, ruthenium isothiocyanate; FITC, fluorescein isothiocyanate; CV, crystal violet; Rh6G, rhodamine 6G; DTDC, 3,3′-diethylthiadicarbocyanine iodide; DTTC, 3,3′-diethylthiatricarbocyanine iodide; UCNP, upconverter nanoparticle; Cy, cyanine; PEG, polyethylene glycol; HNP, hexoctahedral nanoparticle; IR780, iodide dye; R6G, rhodamine 6G; PDA, polydopamine; BSA, bovine serum albumin; EpCAM, epithelial cell adhesion molecule; EGFR, epidermal growth factor receptor; PDDA, polydiallyldimethylammonium chloride; PTK, tyrosine-protein kinase; MPN, metal–phenolic network; P-MPN, plasmonic metal–phenolic network; MG, malachite green; AB, astra blue; PE-eFluor 610, phycoerythrin–eFluor 610.

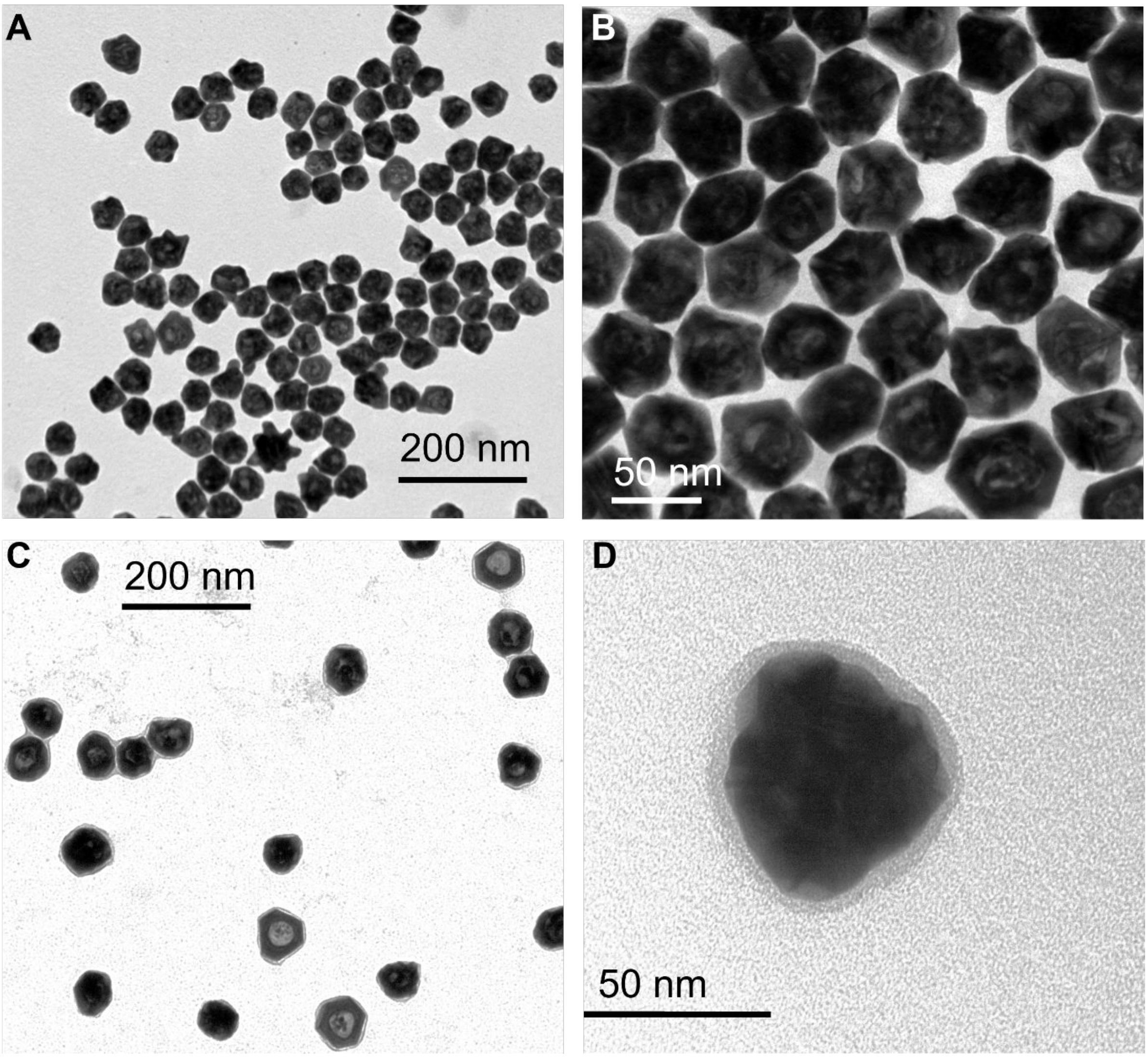

Figure S1. TEM images of Au hollow nanocapsules (A,B) before and (C,D) after coating with MPNs.

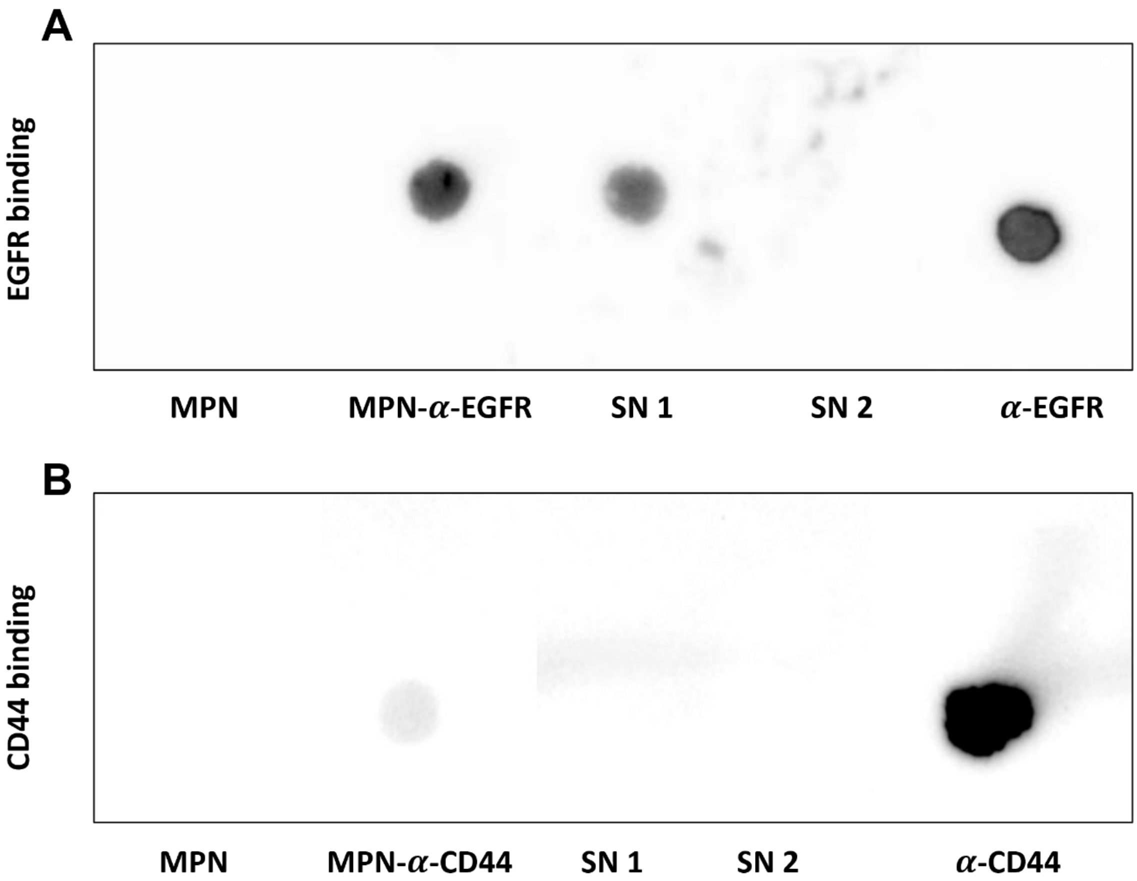

Figure S2. Dot blot analysis of (A) anti-EGFR@FITC and (B) anti-CD44@PE-eFluor 610 antibodies bound to the P-MPN probes. From left to right, samples of the P-MPN without antibodies (MPN), the P-MPN with antibodies (MPN-α-EGFR or MPN-α-CD44), first (SN1) and second (SN2) supernatants, and the pure antibodies (α-EGR and α-CD44) were assessed by anti-HRP recognition through chemiluminescence of the peroxidase.

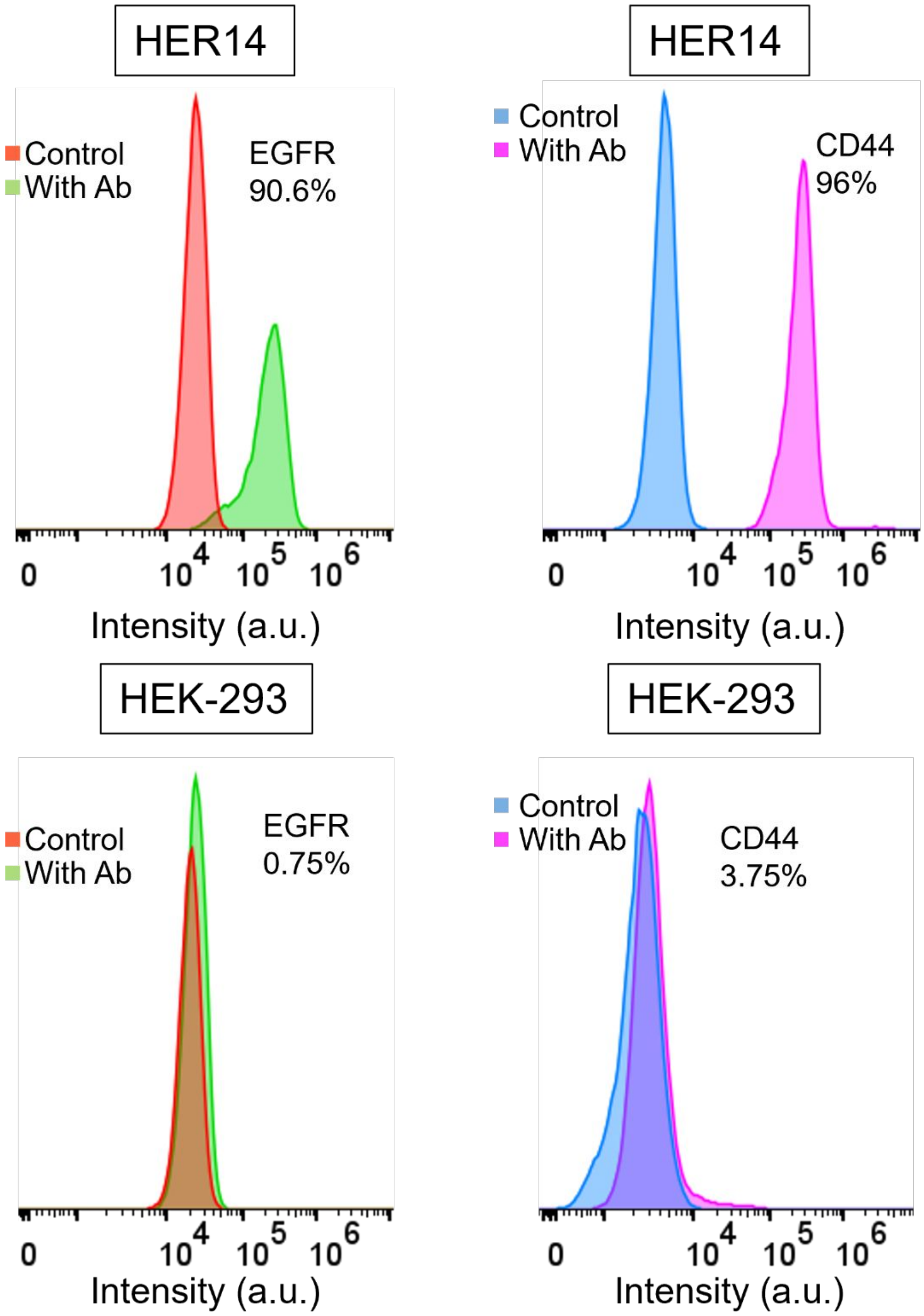

Figure S3. Flow cytometry analysis of the expression of EGFR (left) and CD44 (right) in HER14 (top) and HEK-293 (bottom) cell lines. For HER14 cells, the biomarker expression was 90.6% of EGFR and 96% CD44. For HEK-293 cells, the biomarker expression was 0.75% for EGFR and 3.75% for CD44. The autofluorescence of each cell line was used as a control for each analysis.

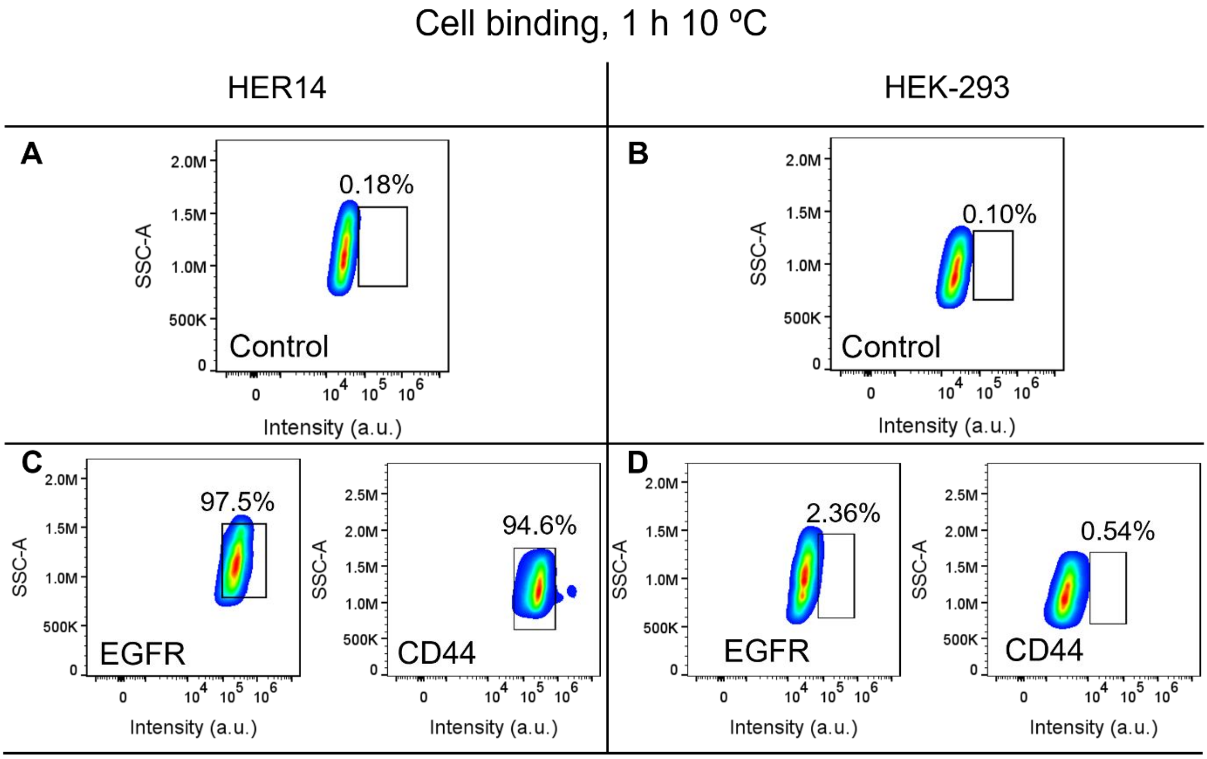

Figure S4. Flow cytograms showing individual binding of the plasmonic nanoparticle dual probes P-MPN_{EGFR} and P-MPN_{CD44} to HER14 and HEK-293 cells. For cell binding studies, the nanoparticles were incubated at 10 °C for 1 h with the cells. (A,B) Control cells without probes. (C,D) Binding of the nanoparticles to EGFR and CD44 in HER14 (C) and HEK-293 (D) cells. The positive percentages for the expression of EGFR or CD44 are shown in each cytogram.

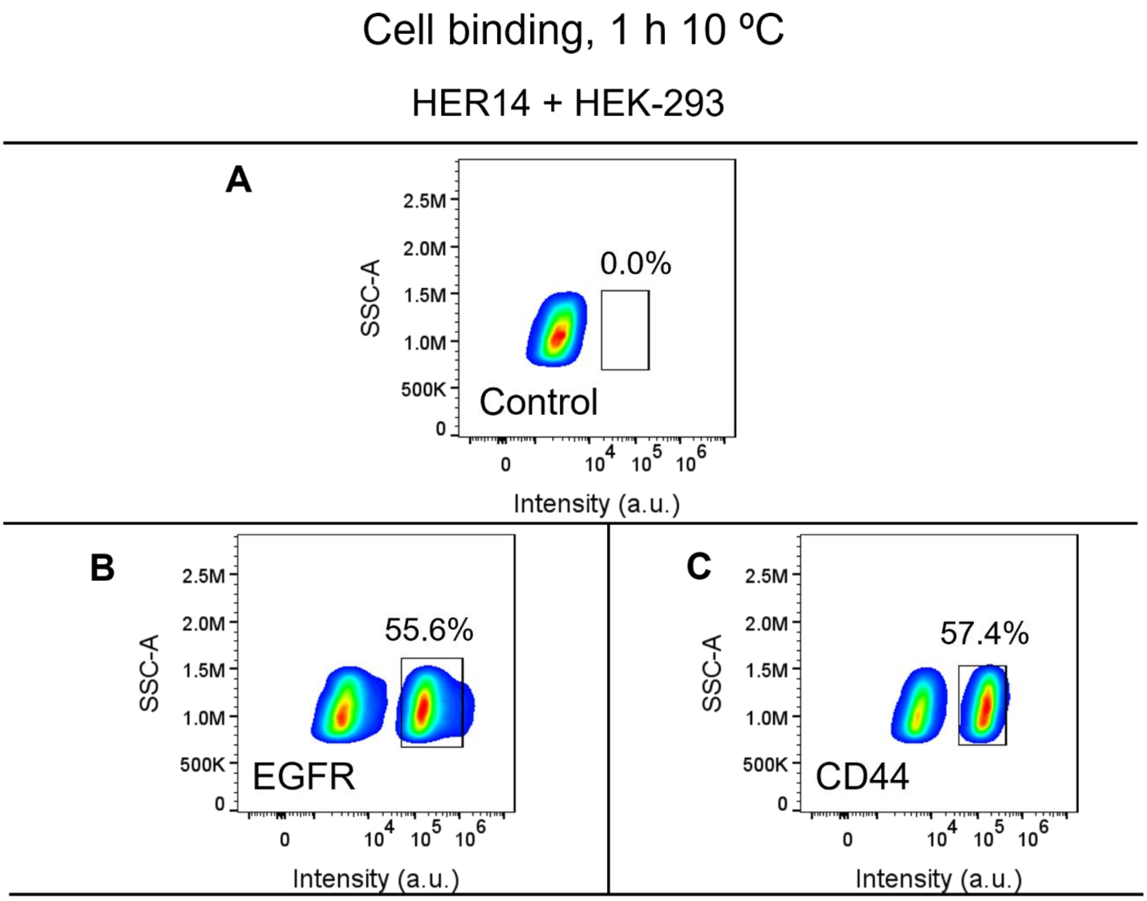

Figure S5. Flow cytograms showing binding of P-MPN$_{EGFR}$ and P-MPN$_{CD44}$ dual probes with HER14 and HEK-293 cells present in a mixed cell culture. For cell binding studies, the nanoparticles were incubated at 10 °C for 1 h with the cells. (A) Control mixed cells without probes. (B,C) Binding of the dual-mode probes to EGFR (B) and CD44 (C) in the mixed culture of HER14 and HEK-293 cells. The percentages of the cells positive for both biomarkers are shown in each cytogram.

References

(1) Kim, H.-M.; Kim, D.-M.; Jeong, C.; Park, S. Y.; Cha, M. G.; Ha, Y.; Jang, D.; Kyeong, S.; Pham, X.-H.; Hahm, E.; Lee, S. H.; Jeong, D. H.; Lee, Y.-S.; Kim, D.-E.; Jun, B.-H. Assembly of Plasmonic and Magnetic Nanoparticles with Fluorescent Silica Shell Layer for Tri-Functional SERS-Magnetic-Fluorescence Probes and its Bioapplications. *Sci Rep* 2018, *8* (1), 13938. https://doi.org/10.1038/s41598-018-32044-7.

(2) Wang, Y.; Chen, L.; Liu, P. Biocompatible Triplex Ag@SiO_2@mTiO_2 Core–Shell Nanoparticles for Simultaneous Fluorescence-SERS Bimodal Imaging and Drug Delivery. *Chem Eur J* 2012, *18* (19), 5935–5943. https://doi.org/10.1002/chem.201103571.

(3) Zhang, X.; Kong, X.; Lv, Z.; Zhou, S.; Du, X. Bifunctional Quantum Dot-Decorated Ag@SiO2 Nanostructures for Simultaneous Immunoassays of Surface-Enhanced Raman Scattering (SERS) and Surface-Enhanced Fluorescence (SEF). *J Mater Chem B* 2013, *1* (16), 2198. https://doi.org/10.1039/c3tb20069h.

(4) Wang, Z.; Zong, S.; Li, W.; Wang, C.; Xu, S.; Chen, H.; Cui, Y. SERS-Fluorescence Joint Spectral Encoding Using Organic–Metal–QD Hybrid Nanoparticles with a Huge Encoding Capacity for High-Throughput Biodetection: Putting Theory into Practice. *J Am Chem Soc* 2012, *134* (6), 2993–3000. https://doi.org/10.1021/ja208154m.

(5) Navas-Moreno, M.; Mehrpouyan, M.; Chernenko, T.; Candas, D.; Fan, M.; Li, J. J.; Yan, M.; Chan, J. W. Nanoparticles for Live Cell Microscopy: A Surface-Enhanced Raman Scattering Perspective. *Sci Rep* 2017, *7* (1), 4471. https://doi.org/10.1038/s41598-017-04066-0.

(6) Zhang, Y.; Wang, Z.; Wu, L.; Zong, S.; Yun, B.; Cui, Y. Dual Peptides Modified Fluorescence-SERS Dual Mode Imaging Nanoprobes with Improved Cancer Cell Targeting Efficiency. *RSC Adv* 2016, *6* (84), 81046–81052. https://doi.org/10.1039/C6RA13802K.

(7) Zong, S.; Wang, Z.; Yang, J.; Wang, C.; Xu, S.; Cui, Y. A SERS and Fluorescence Dual Mode Cancer Cell Targeting Probe Based on Silica Coated Au@Ag Core–Shell Nanorods. *Talanta* 2012, *97*, 368–375. https://doi.org/10.1016/j.talanta.2012.04.047.

(8) Wang, Z.; Zong, S.; Yang, J.; Li, J.; Cui, Y. Dual-Mode Probe Based on Mesoporous Silica Coated Gold Nanorods for Targeting Cancer Cells. *Biosens Bioelectron* 2011, *26* (6), 2883–2889. https://doi.org/10.1016/j.bios.2010.11.032.

(9) Tan, H.; Ou, J.; Hou, Y.; Dai, X.; Yang, Y.; Ma, S.; Chen, X. Surface-Enhanced Raman Scattering-Fluorescence Dual-Mode Probes for Target Imaging of Tumors, Organoids and Cancerous Cells. *Sens Actuators B Chem* 2024, *414*, 135974. https://doi.org/10.1016/j.snb.2024.135974.

(10) Lee, S.; Chon, H.; Yoon, S.-Y.; Lee, E. K.; Chang, S.-I.; Lim, D. W.; Choo, J. Fabrication of SERS-Fluorescence Dual Modal Nanoprobes and Application to Multiplex Cancer Cell Imaging. *Nanoscale* 2012, *4* (1), 124–129. https://doi.org/10.1039/C1NR11243K.

(11) Niu, X.; Chen, H.; Wang, Y.; Wang, W.; Sun, X.; Chen, L. Upconversion Fluorescence-SERS Dual-Mode Tags for Cellular and in Vivo Imaging. *ACS Appl Mater Interfaces* 2014, *6* (7), 5152–5160. https://doi.org/10.1021/am500411m.

(12) Pal, S.; Ray, A.; Andreou, C.; Zhou, Y.; Rakshit, T.; Wlodarczyk, M.; Maeda, M.; Toledo-Crow, R.; Berisha, N.; Yang, J.; Hsu, H.-T.; Oseledchyk, A.; Mondal, J.; Zou, S.; Kircher, M. F. DNA-Enabled Rational Design of Fluorescence-Raman Bimodal Nanoprobes for Cancer Imaging and Therapy. *Nat Commun* 2019, *10* (1), 1926. https://doi.org/10.1038/s41467-019-09173-2.

(13) Hu, Y.; Xu, L.; Miao, X.; Xie, Y.; Zhang, Z.; Wang, Y.; Ren, W.; Jiang, W.; Wang, X.; Wu, A.; Lin, J. SERS/Fluorescence Dual-Modal Imaging Bioprobe for Accurate Diagnosis of Breast Cancer. *Anal Chem* 2025, *97* (10), 5527–5537. https://doi.org/10.1021/acs.analchem.4c05800.

(14) Wang, X.; Yang, H.; Sun, T.; Zhang, J.; Wang, L.; Zhang, Y.; Zhou, N. A Fluorescence and SERS Dual-Mode Biosensor for Quantification and Imaging of Mucin1 in Living Cells. *Biosens Bioelectron* 2025, *270*, 116964. https://doi.org/10.1016/j.bios.2024.116964.

(15) Xiao, L.; Parchur, A. K.; Gilbertson, T. A.; Zhou, A. SERS-Fluorescence Bimodal Nanoprobes for *in Vitro* Imaging of the Fatty Acid Responsive Receptor GPR120. *Anal Methods* 2018, *10* (1), 22–29. https://doi.org/10.1039/C7AY02039B.

(16) Bamrungsap, S.; Treetong, A.; Apiwat, C.; Wuttikhun, T.; Dharakul, T. SERS-Fluorescence Dual Mode Nanotags for Cervical Cancer Detection Using Aptamers Conjugated to Gold-Silver Nanorods. *Microchim Acta* 2016, *183* (1), 249–256. https://doi.org/10.1007/s00604-015-1639-9.
